# SLU long-term field experiments: Soil organic matter in a cereal only cropping system (R3-0020), crop and soil data from 1971 to 2023

**DOI:** 10.1016/j.dib.2026.112817

**Published:** 2026-05-16

**Authors:** Nadia I. Maaroufi, Emme Macdonald, Anke M. Herrmann, Sabina Braun

**Affiliations:** Swedish University of Agricultural Sciences, Department of Soil and Environment, Uppsala SE-750 07, Sweden

**Keywords:** Cereal crop, Yield, Nitrogen fertilization, Straw, Crop residue, Agriculture

## Abstract

The R3–0020 agricultural long-term field experiments investigate the effect of nitrogen addition levels and straw removal on crop yield and soil properties. The field experiments are located at the Swedish University of Agricultural Sciences (SLU) four field research stations: Lönnstorp (Lund), Lanna (Skara), Säby (Uppsala) and Röbäcksdalen (Umeå). The field site in Säby has been maintained since 1970 and the three other sites since 1980. The experiments consist of a cereal only crop rotation, a crop residue treatment (straw removed or incorporated to the soil) and four nitrogen fertilization levels (0, 50, 100 and 150 kg N ha^-1^ yr^-1^). Aboveground crop biomass has been collected annually since the beginning of the experiments (Fig. 1), while topsoil samples were collected 2–8 times in the first 10–20 years and every four year since the 90 s (Fig. 2). We report crop yields and nutrient content for grain and straw, as well as carbon and macronutrient content for soils. The data presented here provides a useful time series for investigating the impacts of cereal-only crop rotation, straw removal and nitrogen fertilization levels on soil organic carbon and yield.

Specifications Table

**Table:** 

Subject	Earth & Environmental Sciences
Specific subject area	Agricultural science, soil science, soil nutrient cycling, and agricultural management practices.
Type of data	Raw table.
Data collection	Crop biomass was harvested at the end of each growing season and split into agronomically-relevant fractions (straw and grain). Soil samples were collected at 0–20 cm depth at various occasions at the start of the experiments and every four year from the 90 s. Soil carbon and macronutrient dry mass content are also reported. All samples were analyzed using standard soil and plant testing protocols at the time of collection. Historical datasets were compiled and digitalized to generate this dataset, and units were harmonized. Data lacking units, corresponding to biologically-infeasible values, or for variables collected only once over the course of the experiment were excluded.
Data source location	The data were collected at Lanna, Lönnstorp, Säby and Röbäcksdalen, Sweden. Archived soil and original data are archived in the Department of Soil and Environment, Swedish University of Agricultural Sciences, Uppsala, Sweden
Data accessibility	Repository name: Researchdata.se, Swedish National Data Service Data identification number: DOI 10.5878/09pa-x212 Direct URL to data: https://doi.org/10.5878/09pa-x212
Related research article	None

## 1 Value of the Data

• This dataset synthesizes 53 years of crop yield, plant nutritional content and soil chemistry from four agronomically relevant locations in Sweden.
• The dataset allows the evaluation of changes in cereal yield, soil fertility, and soil carbon content in response to crop residue removal and different mineral nitrogen application regimes in contrasting pedoclimatic region of Sweden.
• The long duration of the experiments allows development and validation of models describing slow-acting processes.

## 2 Background

This article describes the dataset of the linked repository of the crop and soil data collected from the four sites included in the long-term agricultural experimental series “Soil organic matter in a cereal only cropping system (R3–0020)” between the years 1971 and 2023 [1]. The experimental series was established to determine how a cereal-only crop rotation affects soil organic matter at various levels of nitrogen application and with straw either removed or left on field. The geographical location of the experimental sites also gives information about how the climatic conditions interplay with the tested factors. Furthermore, the R3–0020 series allow useful comparisons with other crop rotation schemes, such as ley-cereal crop rotations typical in the Swedish context (e.g., see R3–0021).

Tackling both the decline in soil organic matter pools and the increasing pressure on crop production requires to better understand the interplay between pedoclimatic conditions, management such as low crop rotation diversity, nitrogen application levels and crop residue treatment at both the short and long-term. The R3–0020 long-term field trials presented here, aim to study the change in soil organic matter stocks and macronutrients in response to contrasting nitrogen application levels and straw removal on cereal only crop rotations. The long-term field trials are composed to four experimental sites established in 1970 for Säby site and in 1980 for Lanna, Lönnstorp and Röbäcksdalen sites.

Results from these field sites have been used to study, e.g., soil organic matter dynamics [2,3] and microbial abundance and activity linked to nutrient cycling [4]. Yet, much of the historical data from each field trial were not digitalized or made available in a data repository, which we address with the present dataset.

## 3 Data Description

Table 1

**Table 1:** Description of the different datasets.

Files	Description
R30020_data.tsv	Plant and soil data as described in README and Fig. 1, Fig. 2, respectively.
R30020_README.txt	Descriptors for each variable presented in subsequent tables
R30020component_description.tsv	Definitions of components listed
R30020_metadata.tsv	Definitions of data columns
R30020unit_description.tsv	Definitions of measurement units
R30020variable_description.tsv	Definitions of variables listed
R30020location_description.tsv	Description of site locations

## 4 Experimental Design, Materials and Methods

### 4.1 Experimental design

**Fig. 1:**
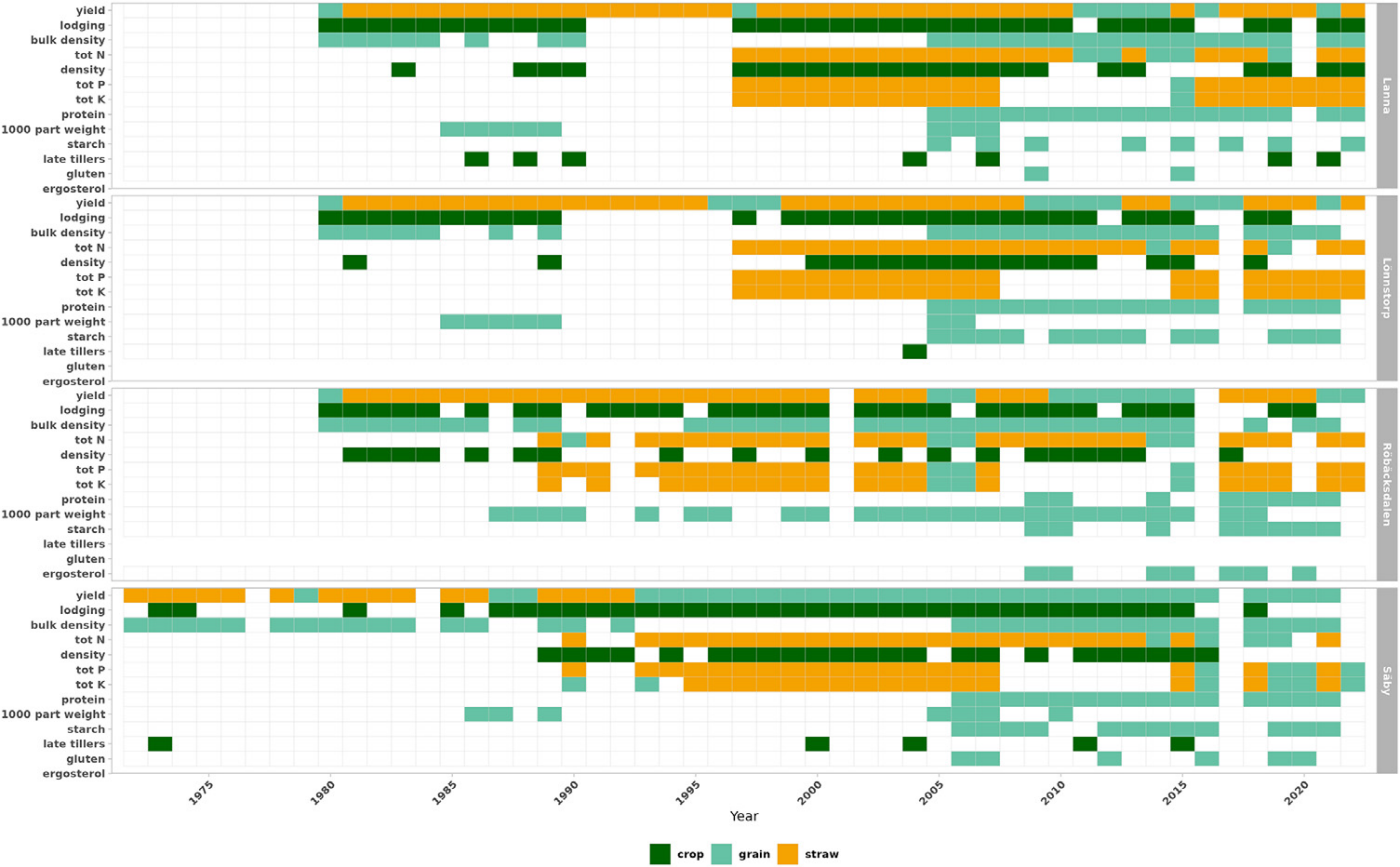
Timeline of availability of crop, grain and straw data by year and sites.

**Fig. 2:**
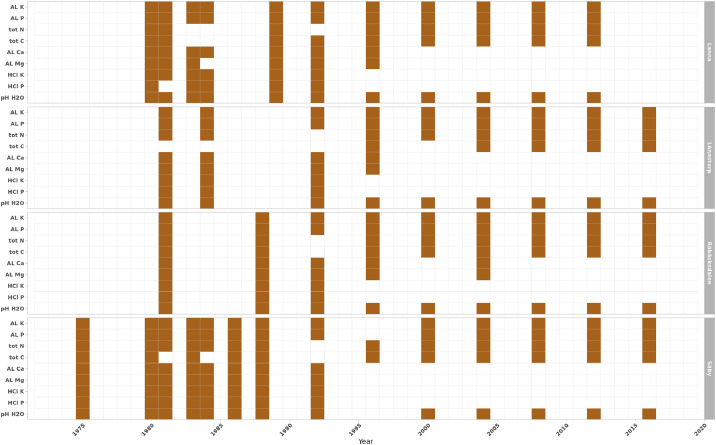
Timeline of availability of soil data by year and sites. HCl: hydrochloric acid extractable nutrients, AL: ammonium-lactate extractable nutrient.

The data described in this article are derived from the R3–0020 experimental series on soil organic carbon first initiated in 1970 in Säby, followed by Lanna, Lönnstorp and Röbäcksdalen in 1980 (Fig. 1, 2). The coordinates and pedoclimatic properties for the sites are given in Table 2.

**Table 2:** Coordinate and pedoclimatic properties of the four sites of R3–0020 trials.

	Lönnstorp	Lanna	Säby	Röbäcksdalen
Coordinate	55.40 °N, 13.05 °E	58.21 °N, 13.08 °E	59.49 °N, 17.42 °E	63.48 °N, 20.13 °E
Soil type^1^	Dystric Cambisol	Eutric Cambisol	Eutric Cambisol	Dystric Cambisol
Average yearly temperature^2^ (⁰C)	8.8	7.3	6.7	4.1
Average yearly precipitation^3^ (mm)	567	558	588	529

1 Poeplau et al., (2015) [2].

2 Normalvärden för temperatur, SMHI, https://www.smhi.se/data/temperatur-och-vind/temperatur/dataserier-med-normalvarden-for-perioden-1991–2020.

3 Climatological normals for 1991–2020, Normalvärden för nederbörd, SMHI, https://www.smhi.se/data/temperatur-och-vind/temperatur/dataserier-med-normalvarden-for-perioden-1991–2020.

The R3–0020 trials consist of a split-plot design experiment with the crop residue treatment as the whole plot factor and N addition treatment as the subplot factor. The crop residue treatment consists of either removing straw or chopping the straw and incorporating it to the ground by ploughing. The N treatment consists of four N addition levels (0, 20, 80 and 120 kg N ha^-1^ yr^-1^) applied in spring as granulates before sowing the 90 m^2^ plots by broadcast application with tractor-mounted spreader. The nitrogen is applied as calcium sulphate ammonium-nitrate (27%N, 9% SO_3_, Ca 0.6%, YaraBela AXAN). The plots are arranged in a randomized block design (four blocks), yielding to a total of 32 plots (four replicates × two crop residue treatments × four N addition levels). The rotation consists of a full cereal crop rotation composed of oat (*Avena sativa* L.), spring barley (*Hordeum vulgare* L.), spring and winter wheat (*Triticum aestivum* L.).

At the first four-year rotation, phosphorus and potassium were applied during every other year in spring at a rate of 500 kg ha^-1^ (P:K, 7:13). Hereafter, the PK fertilizer was applied according to the replacement principle, based on the preceding total P and K outputs of the last rotation. The PK fertilizer is applied at year two and four of the rotation period in spring. All trials were also fertilized with micronutrients. Note that the Säby field site was limed in fall 2017.

### 4.2 Crop and soil monitoring and sampling

In each plot, crop density, number of green shoots, fungal and parasite infection were recorded in spring as well as straw strength during harvest in late summer-early fall. Date of heading was recorded treatment wise.

A total of 10 soil cores were taken per plot using a 2 cm diameter soil corer every four year in autumn (Fig. 2). The soil cores were taken up to 20 cm depth and the first top cm of the soil cores were discarded. The 10 subsamples were subsequently pooled to obtain one composite sample per plot.

### 4.3 Plant and soil analyses

All samples were analyzed at the Soil and Plant Laboratory, Department of Soil and Environment, Swedish University of Agricultural Sciences, Uppsala, Sweden.

At harvest, both grains and straw were weighted before and after drying at 65 °C during 24 h. Crop yield was calculated and expressed in kg ha^-1^. Grain quality was determined by measuring the water, protein, starch, gluten and ergosterol content using the Near-Infrared Transmittance (NIT, FOSS Infractec).

After collection, soil samples were air-dried for a week at ambient temperature and sieved at 2 mm with root and coarse material removed. The pH was determined by shaking the soil sample with water at a soil:water ratio of 1:3.

Total carbon and nitrogen content of plant and soil were measured by dry combustion using an elemental analyzer (TruMac CN, LECO) following the Dumas method. Until 1983, total carbon and nitrogen were analyzed using the Walkley‐Black [5] and Kjeldahl digestion [6] methods, respectively.

Total phosphorus and potassium were measured on ash diluted in HNO_3_. Plant available soil phosphorus (P-AL), potassium (K-AL), calcium (Ca-AL) and magnesium (Mg-AL) were extracted with an ammonium lactate-acetic acid solution. Plant available soil phosphorus (P-HCl) and potassium (K-HCl) were also extracted in a 2 M chlorohydric acid solution. All extracts were measured with an ICP-OES (AVIO 200, Perkin Elmer).

## Limitations

Not applicable.

## Ethics Statement

The authors confirm that they have read and followed the ethical requirements for publication in Data in Brief and confirm that the current work does not involve human subjects, animal experiments, or any data collected from social media platforms.

## Credit Author Statement

**Nadia I. Maaroufi:** Writing – original draft, Writing, Project administration – review & editing, Visualization.

**Sabina Braun:** Data curation, Validation, Investigation, Writing – review & editing, Project administration.

**Emme MacDonald:** Data curation, Validation, Writing.

**Anke M. Herrmann:** Funding acquisition.

## Data Availability

Swedish National Data ServiceSLU long-term field experiments: Soil organic matter in a cereal-only cropping system (R3-0020), crop and soil data from 1971 and onwards (Original data)
